# Acute suppurative thyroiditis with Graves disease – A very rare association

**DOI:** 10.20945/2359-3997000000610

**Published:** 2023-05-29

**Authors:** Inês Damásio, Joana Maciel, Maria Manuel Costa, Luisa Raimundo

**Affiliations:** 1 Instituto Português de Oncologia de Lisboa Francisco Gentil Lisboa Portugal Serviço de Endocrinologia, Instituto Português de Oncologia de Lisboa Francisco Gentil, Lisboa, Portugal; 2 Hospital de Braga Braga Portugal Serviço de Endocrinologia, Hospital de Braga, Braga, Portugal; 3 Hospital Garcia de Orta Almada Portugal Serviço de Endocrinologia, Hospital Garcia de Orta, Almada, Portugal morada

**Keywords:** Graves disease, acute thyroiditis, suppurative thyroiditis, thyrotoxicosis

## Abstract

Acute suppurative thyroiditis is an uncommon disorder caused by a bacterial infection, usually presenting with normal thyroid function. It is a serious condition that requires a prompt diagnosis and treatment with antibiotics and supportive measures. A 62 years-old female presented with a painful cervical induration and odynophagia a week after a fish bone had been removed from her pharynx. She was febrile, and tachycardic and, on physical examination, a painful thyroid mass was detected. High inflammatory parameters and thyrotoxicosis were confirmed: thyroid stimulating hormone (TSH) < 0.01 mIU/L (normal range [NR] 0.27-4.2); free thyroxine (FT4) 3.86 ng/dL (NR 0.9-1.7) and anti-TSH receptor antibodies (TRABs) 5.3 U/L (NR < 1.5). Thyroid scintigraphy showed a diffuse uptake of the thyroid parenchyma suggesting Graves disease. Cervical ultrasonography revealed an abscess of the left thyroid lobe of 36 × 36 mm and fine needle aspiration biopsy (FNAB) with partial drainage was performed. *Staphylococcus aureus* and *Streptococcus viridans* were isolated, and directed antibiotic therapy was started. Clinical improvement was observed as well as a decrease of inflammatory parameters and the patient was discharged after 9 days of hospitalization. Eighteen days after discharge, thiamazole was initiated due to persistent thyrotoxicosis. Complete resolution of the abscess was documented within 6 months and the patient became euthyroid under thiamazole one year after initial presentation. To our knowledge, this is the third case reporting an association between acute thyroiditis and Graves disease. Furthermore, this is the first case detailing the simultaneous diagnosis of acute suppurative thyroiditis caused by a foreign body and Graves disease.

## INTRODUCTION

Acute suppurative thyroiditis (AST) is an uncommon condition that occurs mainly in children and adults with ages ranging between 20 and 40 years old, with a female-to-male ratio of 1:1 (1,2). It is usually caused by a bacterial infection and it is more frequent among immunosuppressed patients or in the setting of pre-existing thyroid disease (3). Anatomical deformities such as pyriform sinus fistula and thyroglossal duct cyst are commonly found in AST. Rarely, it occurs as a complication after fine needle aspiration biopsy (FNAB) or esophageal microperforation (3). Typically, AST presents with anterior neck swelling, pain, and fever (1). Thyroid function is usually normal, but AST can lead to transient thyrotoxicosis due to the destruction of thyroid follicles and release of preformed thyroid hormones (1). In most cases, patients experience complete resolution with antibiotics, however mortality may result from treatment delay (1). Here, we report a case of coexisting AST and Graves disease (GD).

## CASE PRESENTATION

**Figure 1:**
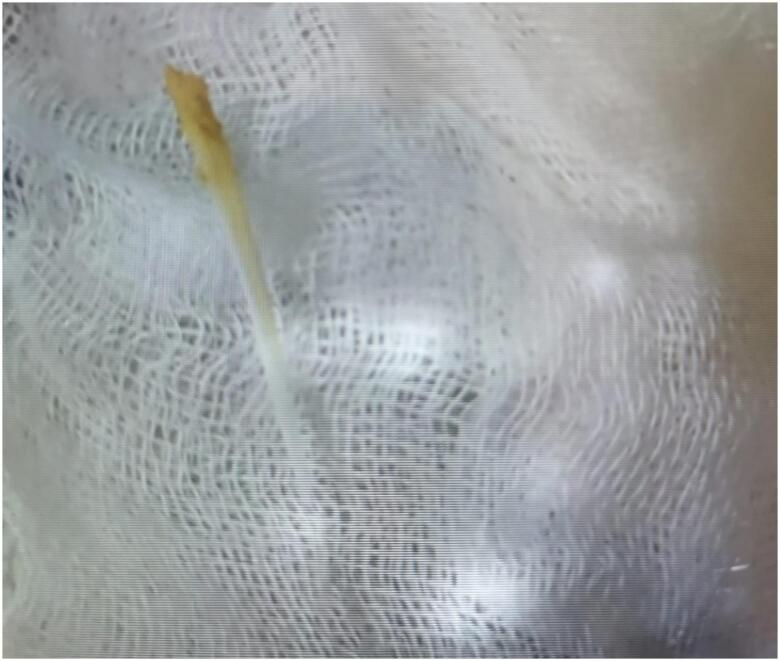
The fish bone removed from hypopharynx measuring 4 cm.

A 62-year-old female with a history of arterial hypertension under treatment with lisinopril presented at the emergency department reporting neck pain that had started shortly after a fish meal. She had no past medical history of thyroid disease. The patient was examined by an ear, nose, and throat specialist and a fish bone of 4 cm was removed from the hypopharynx (Figure 1). The procedure was uneventful and the patient was discharged clinically improved.

One week later, she returned to the emergency department presenting neck swelling associated with local pain and odynophagia. At the time of observation, her body temperature was 38.2 ºC and her heart rate was 103 beats per minute. On physical examination, a painful neck mass was detected and no erythema or other skin changes were observed. Laboratory analysis revealed high inflammatory parameters, thyrotoxicosis (TSH [thyroid stimulating hormone] < 0.01 mIU/L [NR 0.27-4.2]; FT4 [free thyroxine] 3.86 ng/dL [NR 0.9-1.7]) and positive anti-TSH receptor antibodies (TRABs) (5.3 U/L [NR < 1.5]), suggesting GD (Table 1). She was then admitted to the hospital for therapeutic and diagnostic purposes. Treatment with empirical antibiotic (amoxicillin plus clavulanic acid) and corticosteroids (methylprednisolone) was initiated.

**Table 1:** Laboratory analysis during follow-up

	ED	D9	D18 AD	2M AD^1^	5M AD^2^	9M AD^3^	15M AD^4^	Normal range
Leukocytes	9.4		4.0	4.9	4.3	-	-	4.0-11.0 10^9 /L
CRP	15.9	1.44	1.28	2.42	1.46	-	0.1	0.0-0.2 mg/dL
ESR	120	95	66	83		64	21	<31 mm 1º hour
TSH	<0.01	<0.01	<0.01	<0.01	<0.01	0.01	2.12	0.27-4.2 mIU/L
FT4	3.86	3.04	2.22	2.61	1.55	1.43	0.90	90-170 ng/dL
TT3	137	187	360	327	176	-		80-200 ng/dL
TRABs	5.3	--	3.5	5	4.8	3.9	2.7	<1.5 U/L

Hemoglobin, platelets, erythrocytes, hepatic and renal function, sodium and potassium, were always under normal values. Antibodies anti-thyroglobulin and anti-thyroid peroxidase were negative.

1 The patient was under thiamazole 10 mg per day.

2 The patient was under thiamazole 20 mg per day.

3 The patient was under thiamazole 15 mg per day.

4 The patient was under thiamazole 2.5 mg per day.

CRP: C reactive protein; ESR: erythrocyte sedimentation rate; TSH: thyroid stimulating hormone; FT4: free thyroxine; TT3: total triiodothyronine; TRABs: TSH receptor antibodies; ED: emergency department; D9: hospital discharge; AD: after discharge; D: day; M: month.

**Figure 2:**
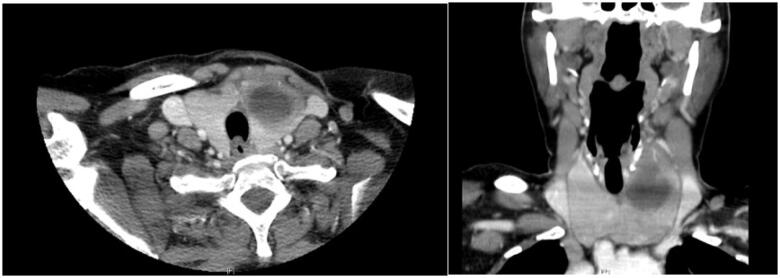
Neck computed tomography scan showing a hypodense nodule of thyroid left lobule measuring 30 × 35 × 35 mm, with tracheal compression.

**Figure 3:**
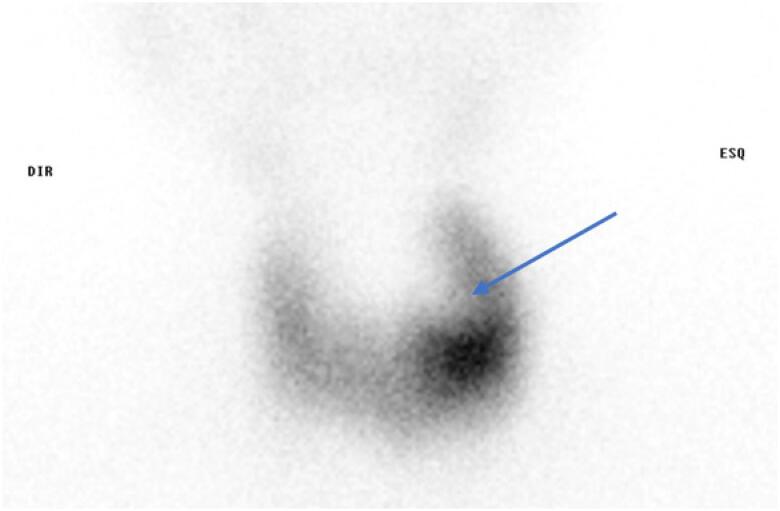
Thyroid scintigraphy with 99mTc-Pertecnetate showing a cold nodule of the left thyroid lobe (blue arrow) surrounded by diffuse uptake of the thyroid parenchyma.

**Figure 4:**
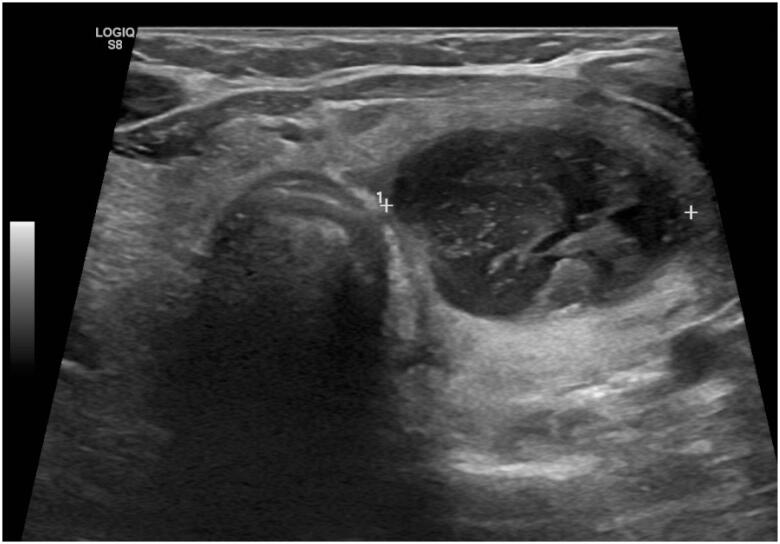
First thyroid ultrasonography performed showing a nodular lesion at left thyroid lobe with a solid central component, well delimited, without hypervascularity measuring 36 × 36 mm.

A neck computed tomography (CT) revealed a large left thyroid nodule measuring 30 × 35 mm (Figure 2). Thyroid scintigraphy with 99mTc-Pertecnetate showed diffuse uptake of the thyroid parenchyma compatible with GD and a left cold nodule (Figure 3). Neck ultrasonography (Figure 4) confirmed the presence of a nodule of the left thyroid lobe with a solid central component, well delimited, without hypervascularity, that was suggestive of an abscess. A FNAB was performed and purulent content was partially aspirated. The cytological analysis revealed several neutrophils and fibrine, small amounts of colloid and no follicular epithelium, suggestive of acute thyroiditis. Cultures of tissue obtained by FNAB were positive for *Staphylococcus aureus* and *Streptococcus viridans* and directed antibiotic therapy was implemented with clindamycin.

During hospitalization, clinical improvement was observed as well as a decrease in inflammatory markers but thyrotoxicosis persisted at day 5 (Table 1). After 9 days, a normalization of inflammatory parameters was noticed (Table 1) and the patient was discharged with the indication to continue clindamycin and corticosteroid therapy at home. However, 18 days after discharge, laboratory analysis revealed persistent thyrotoxicosis and thiamazole was initiated, being adjusted after that according to the patient status and the laboratory findings (Table 1).

Two months after discharge, the patient reported poor compliance to antibiotic therapy, with several omitted doses. At that time, laboratory tests revealed a new rise in inflammatory parameters (Table 1). The patient was then re-admitted to the daycare hospital, where she completed 28 days of intravenous clindamycin. Serial thyroid ultrasonographies were performed, showing a diffusely heterogeneous and large thyroid gland, with features of thyroiditis. At the end of antibiotic course, a residual liquid collection measuring 9 × 4 x 7 mm in left thyroid lobe was identified. Six month after initial presentation, complete resolution of the thyroid abscess was documented by ultrasound.

Corticosteroid therapy was tapered throughout the follow-up and thyroid function control occurred 12 months after presentation with a daily dose thiamazole dose of 2.5 mg. This dose was maintained until the last medical visit (at 15 months of follow-up), and the patient was still euthyroid. There was a gradual decrease in TRABs titters and a minimum value of 2.7 U/L was reached at the end of follow-up.

## DISCUSSION AND CONCLUSION

Suppurative thyroiditis is usually caused by bacterial infection, but fungal, mycobacterial or parasite infections may also occur (2). The reported incidence of AST ranges between 0.1%-0.7% of all thyroid diseases (4). The median age at diagnosis is 40 years, and it occurs equally in both sexes, although some studies show a slight female predominance (4). It is most likely to occur in patients with preexisting thyroid disease (thyroid cancer, Hashimoto's thyroiditis, or multinodular goiter), those with congenital anomalies such as a pyriform sinus fistula (the most common source of infection in children), and those who are immunosuppressed, elderly, or debilitated. Patients with acquired immunodeficiency syndrome are particularly susceptible (2).

To our knowledge, there are only two cases reports of acute thyroiditis and GD. In 1997, Li and cols. reported a case of a diabetic woman with GD in whom thyrotoxicosis occurred after *Klebsiella pneumoniae* thyroiditis (5). Later, in 2019 Lhamu and cols. described a case of a young man with an infected left fourth branchial cleft cyst and GD (6). Here we report the third case associating both conditions.

In our patient's case, no anatomical defects were found. However, there were two causes for thyroid inflammation that could explain the clinical presentation: the perforation by the fish bone and GD. It is well known that the thyroid gland is resistant to infection because of its encapsulation, high iodine content, rich blood supply, and extensive lymphatic drainage (2). We postulate that the concomitant GD in our patient increased the susceptibility to a bacterial spread due to thyroid gland hypervascularity. The location of the suppurative abscess in the left thyroid lobe is not surprising, given the greater susceptibility of this lobe to infections, compared to the right lobe. This is due to the asymmetrical development of the fourth branchial arch (7).

The symptoms and signs reported by our patient illustrate the typical presentation of acute thyroiditis. Most patients with AST present with fever, neck pain and swelling and elevated acute phase reactants (6). AST is associated with an elevation of erythrocyte sedimentation rate (ESR) and C reactive protein (CRP) levels, as showed in our case (8).

Most of patients with AST have normal thyroid function tests. However, there have been few cases reporting thyrotoxicosis in this setting (1,3,8). Thyrotoxicosis is usually transient and results from inflammation and destruction of the gland, with release of preformed thyroid hormones into circulation, in the absence of elevated thyroid antibodies (6,8). However, in this case AST was characterized by excessive discharge of thyroid hormone and elevated TRABs levels, confirming a concomitant GD. The diffuse uptake in thyroid scintigraphy sustains this diagnosis. However, our patient did not have previous symptoms of hyperthyroidism, making reasonable to assume that the acute inflammatory process may have contributed to the exacerbation of thyrotoxicosis by increasing thyroid hormone release.

The most common causative bacteria of AST are *Streptococcus pyogenes* and *Staphylococcus aureus*, responsible for 39% of the reported cases (1). We present a case in which the causative bacteria were *Streptococcus viridans* and *Staphylococcus aureus*. Gram-negative bacilli, anaerobes, tubercle bacillus and fungi can also be implicated, and polymicrobial infection have been reported in about 30% of cases (1).

Despite its rarity, AST is a serious condition with a mortality rate of 3.7%-9% and a timely diagnosis is crucial (4). The mainstay treatment is broad-spectrum antibiotics and drainage is sometimes needed (2,6). However, the treatment course can be long and surgical resolution is required in cases of relapse despite medical treatment (4). A recent review of 2021 by Lafontaine and cols. showed that 32% of the cases of bacterial AST were managed with antibiotics and a single needle aspiration, 3% required multiple needle aspirations and 13% were treated with needle aspiration and antibiotics but subsequently required surgery. The median duration of antibiotic therapy was 17 days (4).

The thyrotoxicosis should be treated with supportive measures (9). In this setting, beta blockers are useful for symptom control, especially in older patients and in those with cardiovascular disease (10). Unlike subacute thyroiditis, glucocorticoid therapy may not be useful in AST or result in only transient responses (4). On the other hand, it is well known that the goal of GD treatment is symptom control and resolution of hyperthyroidism with radioactive iodine ablation, antithyroid drugs or surgery (9).

In our case, the patient started treatment with corticosteroids and antibiotics simultaneously. As expected, the thyrotoxicosis did not resolve with this approach and antithyroid drugs (thionamides) were also required to control thyroid function, as a concomitant autoimmune process was in progress.

We postulated some explanations for the rare association presented in this clinical report. However, the co-existence of AST and GD may be coincidental. Further studies are needed to investigate the relationship between acute bacterial infection of the thyroid and the development of thyroid autoimmunity.
